# Clemastine improves electrophysiologic and histomorphometric changes through promoting myelin repair in a murine model of compression neuropathy

**DOI:** 10.1038/s41598-021-00389-1

**Published:** 2021-10-22

**Authors:** Jung Il Lee, Jong Woong Park, Kyung Jun Lee, Duk Hee Lee

**Affiliations:** 1 Department of Orthopedic Surgery, Korea University Guro Hospital, 148, Gurodong-ro, Guro-gu, Seoul, 08308 South Korea; 2 Department of Orthopedic Surgery, Korea University Anam Hospital, Seoul, South Korea; 3 Department of Orthopedic Surgery, Hanyang University Guri Hospital, Guri, South Korea; 4 Department of Emergency Medicine, Ewha Women’s University Mokdong Hospital, Seoul, South Korea

**Keywords:** Neuroscience, Diseases, Medical research, Neurology

## Abstract

Compression neuropathies are common and debilitating conditions that result in variable functional recovery after surgical decompression. Recent drug repurposing studies have verified that clemastine promotes functional recovery through enhancement of myelin repair in demyelinating disease. We investigated the utility of clemastine as a treatment for compression neuropathy using a validated murine model of compression neuropathy encircling the compression tube around the sciatic nerve. Mice received PBS or clemastine solution for 6 weeks of compression phase. Mice taken surgical decompression received PBS or clemastine solution for 2 weeks of decompression phase. Electrodiagnostic, histomorphometric, and Western immunoblotting analyses were performed to verify the effects of clemastine. During the compression phase, mice treated with clemastine had significantly decreased latency and increased amplitude compared to untreated mice that received PBS. Histomorphometric analyses revealed that mice treated with clemastine had significantly higher proportions of myelinated axons, thicker myelin, and a lower G-ratio. The expression levels of myelin proteins, including myelin protein zero and myelin associated glycoprotein, were higher in mice treated with clemastine. However, the electrophysiologic and histomorphometric improvements were observed regardless of clemastine treatment in mice taken surgical decompression. Mice treated with clemastine during compression of the sciatic nerve demonstrated that clemastine treatment attenuated electrophysiologic and histomorphometric changes caused by compression through promoting myelin repair.

## Introduction

Compression neuropathy or peripheral nerve compression syndrome, such as carpal tunnel syndrome, cubital tunnel syndrome, and tarsal tunnel syndrome, are common disorders of the peripheral nervous system (PNS), and are increasing in frequency. Although surgical decompression of the compressed nerve can be successful in many patients, especially those with early stage compression, the postoperative outcome may be unpredictable and the recovery of motor function is usually limited even after proper surgical management in later-stage compression^1^. Recent studies report that the main pathophysiology of compression neuropathy is loss of myelin, with axonal degeneration occurring in the later stage of the disease^1–3^. Although surgical decompression is the mainstay of treatment to reverse the pathologic changes in the compressed nerve, therapeutic approaches focusing on the recovery or protection of myelin could be beneficial in some patients with later stage compression neuropathy.

Clemastine is a first-generation antihistamine medication used to treat allergic conditions. Recent drug screening to promote remyelination using micropillar arrays identified clemastine to promote differentiation of oligodendrocyte progenitor cells (OPCs) to mature oligodendrocytes^4^. In vivo studies in preclinical animal models of demyelinating brain disease also showed that clemastine promotes remyelination and improves functional recovery^5–8^. Evidence suggests that this remyelination effect of clemastine is mediated by off-target antimuscarinic action at the muscarinic acetylcholine receptor on OPCs^6,9^. Schwann cells are the main glial cells in the PNS and also express M1, M2, M3, and M4 muscarinic receptors^10,11^. Clemastine is expected to promote Schwann cell-mediated myelin repair, as in previous reports of central nervous system (CNS) demyelinating disease.

Based on previous promising results of clemastine on remyelination in the CNS, we hypothesized that clemastine would (1) improve electrophysiologic and histomorphometric parameters of compression during active compression and (2) attribute to improvement of these parameters after surgical decompression using a murine model of compression neuropathy.

## Results

### Clemastine treatment decreases the latency of impulse conduction and increases CMAP amplitude

**Figure 1:**
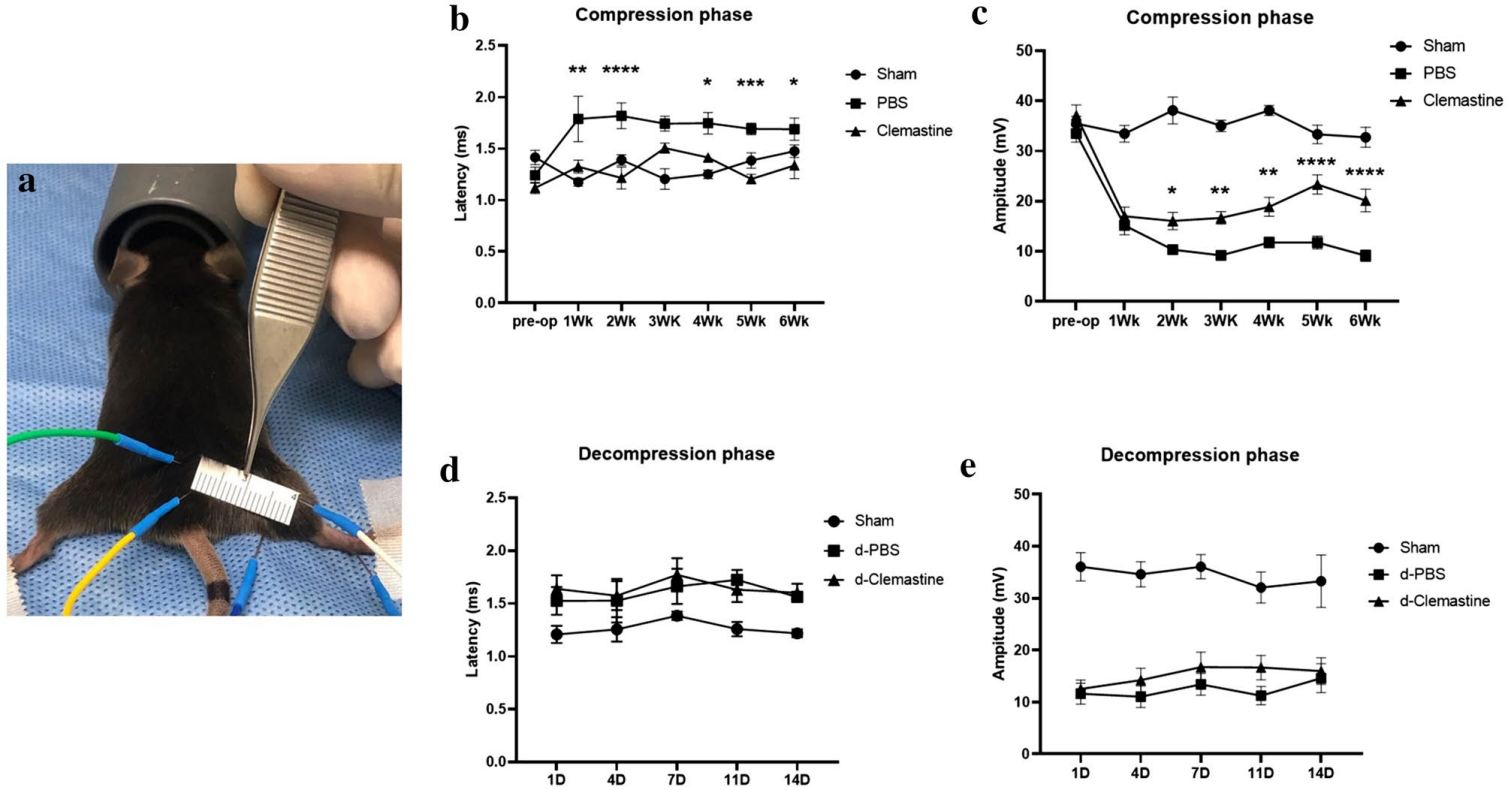
Electrodiagnostic studies to measure latency and amplitude (**a**) (Sham, sham-operated mice; PBS, untreated mice during compression phase; CF, mice treated with clemastine during compression phase; d-PBS, untreated mice after surgical decompression; d-CF, mice treated with clemastine after surgical decompression). Serial measurements of latency and CMAP amplitude during 6 weeks of compression phase in mice with compression neuropathy (**b**,**c**) and during 2 weeks of decompression phase in mice taken surgical decompression (**d**,**e**). Error bars represent standard error of mean. Two-way ANOVA with Turkey’s post-hoc test was conducted. A significant difference (*p < 0.05, **p < 0.01, ***p < 0.001, and ****p < 0.0001) versus PBS (control) or d-PBS group (n = 10 animals/group).

Electrodiagnostic studies to measure latency and compound muscle action potential (CMAP) amplitude were employed to determine the effect of clemastine treatment in compression neuropathy because of their clinical utility in diagnosis and evaluation of compression neuropathy^12,13^. The studies were performed every week during 6 weeks of compression phase and every 3 days during 2 weeks of decompression phase in mice taken surgical decompression. The latency measurements for the untreated mice during the compression phase (PBS group) were delayed compared with sham-operated mice (sham group), and the CMAP amplitude for the untreated mice during the compression phase gradually declined from week 1 through the end of the compression phase (Fig. 1). Clemastine treatment shortened the latency of impulse conduction and increased CMAP amplitude during the compression phase (PBS vs. CF group; Fig. 1b,c). However, the latency and amplitude of clemastine-treated mice during decompression phase (d-CF group) were not significantly different from those of control mice (d-PBS group) (Fig. 1d,e). These findings suggest that clemastine treatment rescues electrophysiologic abnormality caused by compression neuropathy, but these positive effects of clemastine treatment were not observed in mice taken surgical decompression.

### Clemastine treatment improves the ratio of P0-/NF-expressing axons

**Figure 2:**
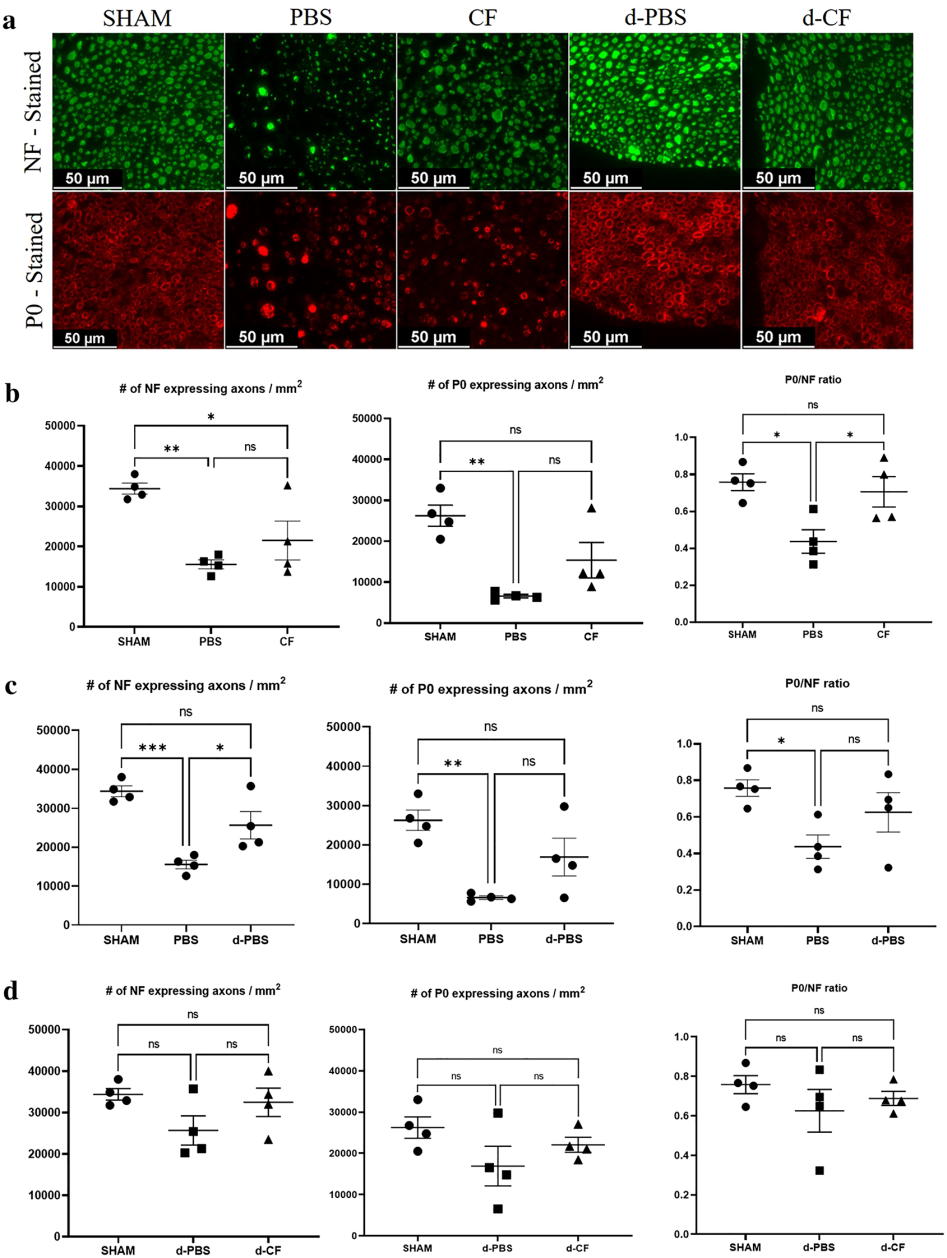
Representative cross-sectional images of the sciatic nerve upon immunofluorescence staining in the compression and decompression phase (**a**) (×400; scale bar = 50 μm; green, NF (neurofilament) heavy chain; red, P0 (myelin protein 0); Sham, nerves of sham-operated mice; PBS, nerves of untreated mice during compression phase; CF, nerves of mice treated with clemastine during compression phase; d-PBS, nerves of untreated mice after surgical decompression; d-CF, nerves of mice treated with clemastine after surgical decompression). Comparison of the number of NF- or P0-expressing axons/mm^2^ and the ratio of P0/NF-expressing axons among sham, PBS, and CF group in the compression phase (**b**). Comparison of these measurements among sham, PBS, and d-PBS group for evaluation of decompression effect (**c**). Comparison of these measurements among sham, d-PBS, and d-CF group in decompression phase (**d**). Error bars represent standard error of mean. One-way ANOVA with Turkey’s post-hoc test was conducted (*p < 0.05, **p < 0.01, and n = 4 animals/group).

To assess the effect of clemastine on the axonal regeneration and myelination, the number of total or myelinated axons was quantified in the cross-sectioned nerve immunostaining with anti-neurofilament (NF) heavy chain and anti-myelin protein zero (P0) antibody (Fig. 2a). The number of NF or P0-expressing axons and ratio of P0-expressing axons/NF-expressing axons for untreated mice during compression phase (PBS group) were decreased compared to those of sham-operated mice (sham group). There was no significant difference in the number of NF- or P0-expressing axons between the untreated mice during the compression phase (PBS group) and clemastine-treated mice during the compression phase (CF group) (NF-expressing axons, 15,563 ± 1121 vs. 21,508 ± 4833, *p* = 0.37; P0-expressing axons, 6587 ± 441 vs. 15,364 ± 4332, *p* = 0.14; Fig. 2b). However, the clemastine-treated mice during the compression phase (CF group) had a significantly higher ratio of P0-expressing axons/NF-expressing axons compared with untreated mice during the compression phase (PBS group) (0.43 ± 0.06 vs. 0.71 ± 0.08, *p* < 0.05). Untreated mice taken surgical decompression(d-PBS group) had a higher number of NF-expressing axons compared to that seen in untreated mice during compression group (PBS group) (15,563 ± 1121 vs. 25,669 ± 3526, *p* < 0.05; Fig. 2c). However, clemastine treatment during decompression phase did not improve the number of NF- or P0-expressing axons and ratio of P0-expressing axons/NF- expressing axons (d-PBS vs. d-CF group; Fig. 2d).

### Clemastine treatment increases the myelin thickness and improves G-ratio

**Figure 3:**
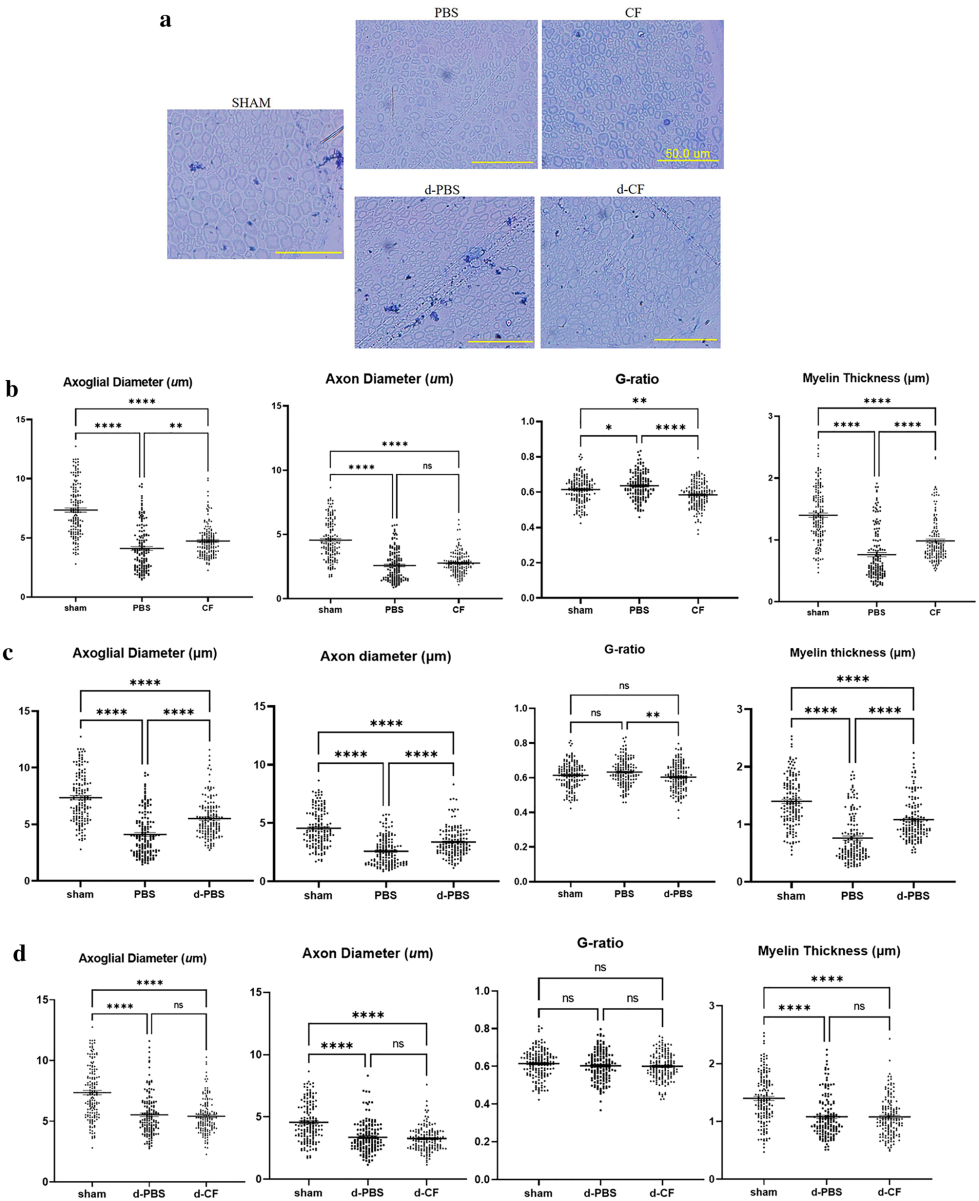
Representative cross-sectional images of the sciatic nerve upon toluidine blue staining in the compression and decompression phase (**a**) (× 400; scale bar = 50 μm; Sham, nerves of sham-operated mice; PBS, nerves of untreated mice during compression phase; CF, nerves of mice treated with clemastine during compression phase; d-PBS, nerves of untreated mice after surgical decompression; d-CF, nerves of mice treated with clemastine after surgical decompression). Comparison of the axoglial diameter, the axonal diameter, the G-ratio and the myelin thickness among sham, PBS, and CF group in compression phase (**b**). Comparison of these measurements among sham, PBS, and d-PBS group for evaluation of decompression effect (**c**). Comparison of these measurements among sham, d-PBS, and d-CF group in decompression phase (**d**). Error bars represent standard error of mean. One-way ANOVA with Turkey’s post-hoc test was conducted (*p < 0.05, **p < 0.01, ***p < 0.001, ****p < 0.0001, and n = 300 axons from 3 animals per group).

While our findings of immunofluorescent staining of cross-sectioned nerves showed that clemastine treatment has beneficial effect of increasing the ratio of P0-expressing axons/NF-expressing axons in compression neuropathy, we further investigated the ultrastructural features of the nerve such as myelin thickness and g-ratio (axonal diameter/axoglial diameter) to confirm the effect on myelination. Figure 3 demonstrates the myeloprotective and/or regenerative effect of clemastine treatment in the compression phase. Axonal diameter, axoglial diameter and myelin thickness in untreated mice during compression phase (PBS group) were decreased compared to those of sham-operated mice (sham group). Mice treated with clemastine during the compression phase (CF group) had a significantly greater axoglial diameter (4.1 ± 0.16 vs. 4.7 ± 0.11 μm, *p* < 0.01), a thicker myelin (0.76 ± 0.03 vs. 0.98 ± 0.03 μm, *p* < 0.001) and a lower G-ratio (0.63 ± 0.01 vs. 0.58 ± 0.01, *p* < 0.001) than the untreated mice during compression group (PBS group) (Fig. 3b). Untreated mice taken surgical decompression (d-PBS group) had a significantly greater axoglial (4.1 ± 0.16 vs 5.5 ± 0.14 μm, *p* < 0.0001), axon diameter (2.6 ± 0.1 vs 3.4 ± 0.1 μm, *p* < 0.0001), a thicker myelin (0.8 ± 0.03 vs 1.1 ± 0.03 μm, *p* < 0.0001) and a lower G-ratio (0.63 ± 0.01 vs 0.6 ± 0.01 μm, *p* < 0.001) than those of untreated mice during the compression phase (PBS group) (Fig. 3c). However, there were no significant differences in these measurements between the two groups (d-PBS vs. d-CF group) in mice taken surgical decompression (Fig. 3d).

### Clemastine treatment increases the expression of myelin protein

**Figure 4:**
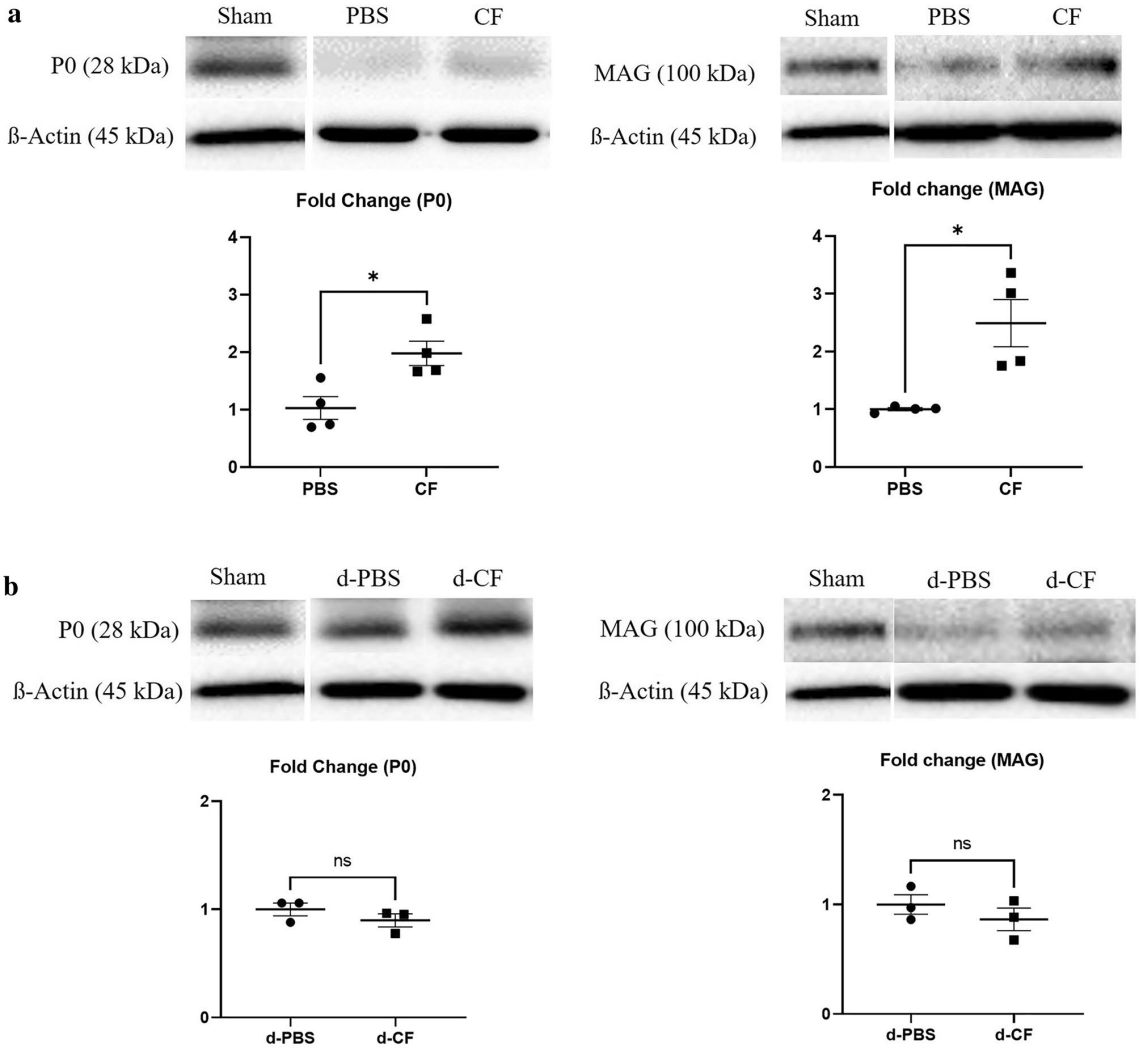
Representative Western immunoblotting for P0 and MAG protein in compression phase (**a**) and decompression phase (**b**) (P0, myelin protein 0; MAG, myelin associated glycoprotein; Sham, nerves of sham-operated mice; PBS, nerves of untreated mice during compression phase; CF, nerves of mice treated with clemastine during compression phase; d-PBS, nerves of untreated mice after surgical decompression; d-CF, nerves of mice treated with clemastine after surgical decompression). Full-length Western blots are presented in Supplementary Figs. 1 and 2. Band intensity quantification of P0 and MAG of CF group as fold change of PBS group. Band intensity quantification of P0 and MAG of d-CF group as fold change of d-PBS group. ß-actin was used to normalize the intensity of the bands. Error bars represent standard error of mean. Mann–Whitney U test was conducted (*p < 0.05, and n = 3 or 4 animals per group).

To determine the clemastine effect on myelination, Western blot was used to quantify the myelin related protein including P0 and myelin-associated glycoprotein (MAG) in the sciatic nerve. Figure 4 shows the Western blot of MAG and P0 expression in sciatic nerves at 6 weeks after nerve compression (Fig. 4a) and 2 weeks after nerve decompression in mice taken surgical decompression (Fig. 4b). The mice treated with clemastine during compression phase (CF group) had higher P0 (1 ± 0.19 vs. 2.0 ± 0.21, *p* < 0.05) and MAG (1 ± 0.03 vs. 2.5 ± 0.41, *p* < 0.05) expression compared to the untreated mice during the compression phase (PBS group) (Fig. 4a). However, these differences in protein levels were not detected between two groups (d-PBS vs. d-CF group) in mice taken surgical decompression.

## Discussion

Sustained administration of clemastine in a murine model of compression neuropathy provides the possibility of a new use for this therapeutic agent, similar to the recent novel use of this agent for remyelination in CNS demyelinating diseases. Here, we demonstrated that sustained clemastine treatment improves electrophysiological parameters, the ratio of P0-/NF-expressing axons, myelin thickness, G-ratio, and myelin protein expression levels in compression neuropathy. However, clemastine treatment did not improve electrophysiologic and histomorphometric parameters in mice taken surgical decompression from compression neuropathy.

Recent high-throughput screening studies demonstrated that muscarinic antagonists such as benztropine, solifenacin, and clemastine promote OPC differentiation and myelination in demyelinating disease^4,9,14^. Among these, clemastine is the most effective in promoting differentiation and myelination of oligodendroglia^4^. Further, clemastine has a known safety profile for clinical use. Pre-clinical in vivo studies validated the myelinating efficacy of clemastine in a lysolecithin-induced demyelinating model and a cuprizone murine model of demyelination^4,5^. Additional studies confirmed the functional benefits of clemastine through enhancing myelin repair in a murine model of depression and hypoxic brain damage^7,8^. A recent clinical trial with clemastine in patients with multiple sclerosis is noteworthy^15^. In this study, clemastine administration for 60 days reduced visual-evoked potential P100 latency and improved low-contrast letter acuity in patients with relapsing multiple sclerosis with chronic demyelinating optic neuropathy. The results of this clinical trial show electrophysiological evidence of remyelination and functional (visual) improvement after clemastine treatment.

The pathophysiology of compression neuropathy is not completely understood, but recent evidence suggests that this injury is Schwann cell-mediated disease^1–3^. Extra-neural compression interferes with the microcirculation of nerves, which in turn can lead to edema formation within the nerve. Subsequently, this process results in perineural fibrosis around the nerve, persistently increasing intraneural pressure^16^. Mechanical stress induces concurrent apoptosis and proliferation of Schwann cells, which are represented by localized segmental demyelination and remyelination^1–3^. With increased compression pressure or duration, there will be increased diffuse demyelination and axonal degeneration. Thus, therapeutic approaches targeting Schwann cell-mediated remyelination could be a reasonable option to improve function in patients with compression neuropathy.

Evidence suggests that OPCs express all subtypes of muscarinic receptors (M1–M5)^17,18^. On the basis of previous studies of action mechanisms, antagonism of M1 and/or M3 subtype of muscarinic receptors expressed on OPCs is responsible for the effect of remyelination after admiration of muscarinic antagonists^6,9^. Schwann cells are the main glial cells in the PNS and play an essential role in the insulation of axons by forming myelin, resulting in fast propagation of action potentials. Schwann cells also express the M1–M4 subtypes of muscarinic receptors on their membrane^10,11^. The muscarinic receptor type expressed on Schwann cells could differently mediate Schwann cell proliferation through M1 and/or M3 or differentiation, but mainly through the M2 subtype^17,19,20^. These receptor expression data support the suggestion that muscarinic receptor antagonists contribute to remyelination by Schwann cell proliferation and differentiation into myelinating forms. Furthermore, a recent study showed that M1 receptor antagonist promote neurite outgrowth by enhancement of mitochondrial function and improve functional recovery in rodent models of metabolic-, chemical-, and HIV-related peripheral neuropathy^21^.

Our interest in clemastine for the treatment of compression neuropathy stemmed from the favorable results of preclinical studies and clinical trials of using the said agent in CNS demyelinating disease. In our study, clemastine increased the ratio of P0-/NF-expressing axons, myelin thickness, and myelin protein expression which supports the idea that clemastine could increase myelination in compression peripheral nerve neuropathy similar to its effects on CNS demyelinating disease. These findings correlate with improvement of electrophysiological parameters and suggest that clemastine treatment might alleviate the effects of nerve entrapment. Another possible mechanism of the protective effects observed myelin in this study is anti-inflammatory properties of clemastine. Although main cause of diffuse myelination and axonal degeneration in compression neuropathy has been known to extra- and intra-neural compression, they are often accompanied by inflammatory reactions^22^. Clemastine reduces the accumulation and activation of inflammatory cells by down-regulation of NF-ĸB activation and reducing the synthesis of pro-inflammatory factors^23,24^. The safety profile of clemastine is well-characterized, having over 60 years of clinical use for allergic conditions. We believe that the therapeutic application of clemastine, as suggested by the current study, could have great potential for rapid clinical application.

Electrodiagnostic studies are critical methods for the diagnosis, classification of severity, and prediction of recovery in compression neuropathy^12,13^. Typical electrodiagnostic findings in the initial stage of the disease include an increase in latency and a decrease in nerve conduction velocity, which implies focal demyelination^3,25,26^. As the injury becomes more severe, CMAP amplitude decreases, which implies axonal damage. According to our electrodiagnostic findings of decreased latency and amplitude, our rodent model represents an advanced stage of compression neuropathy. Our results showed that clemastine treatment decreased latency and increased CMAP amplitude, indicating that clemastine enhanced myelination and axonal regeneration in the advanced stage of compression neuropathy.

Our study has several limitations. First, although we demonstrated that clemastine treatment seems to increase myelination through electrodiagnostic and histomorphometric analyses, we cannot know if improvements in these measurements, such as latency, amplitude of the conduction impulse, the ratio of P0-/NF-expressing axons, the myelin thickness, and the G-ratio will translate into clear functional gains. Future studies could focus on the correlation between the positive effects of clemastine and functional improvements after treatment. Moreover, another limitation of our study is that the beneficial effects of clemastine were observed only in the compression phase. There were no differences between the untreated and clemastine-treated mice that underwent decompression surgery. It is presumed that this indistinction is due to the insufficiency of the study’s observational period to reveal the adjuvant effect on myelin repair of clemastine treatment. Other assumption is that surgical decompression masks the effect of clemastine effect, because surgical decompression has much effective in myelin repair than clemastine treatment. Further studies are necessary to investigate the possibility of clemastine treatment as an adjuvant therapy for surgical treatment.

Our results showed that clemastine treatment attenuates the electrophysiological and pathologic changes in nerve tissue caused by compression neuropathy, which further indicates that clemastine promotes myelin repair. These findings suggest the clinical potential of using clementine as a therapeutic agent in the advanced stages of compression neuropathy.

## Materials and methods

### Mouse models of compression neuropathy and surgical decompression

The experimental design and surgical protocols were approved by the Institutional Animal Care and Use Committee (IACUC) in our institution. The mice were housed at the animal facility and handled according to the IACUC guidelines for the care and use of laboratory animals. Ten-week-old female C57BL/6J mice weighing 20–25 g underwent peripheral nerve compressive surgery in which a compressive tube was placed on the right sciatic nerve (n = 40). The sham-operated mice underwent a similar operation in which the sciatic nerve was exposed and isolated but not encircled with a tube (n = 10).

**Figure 5:**
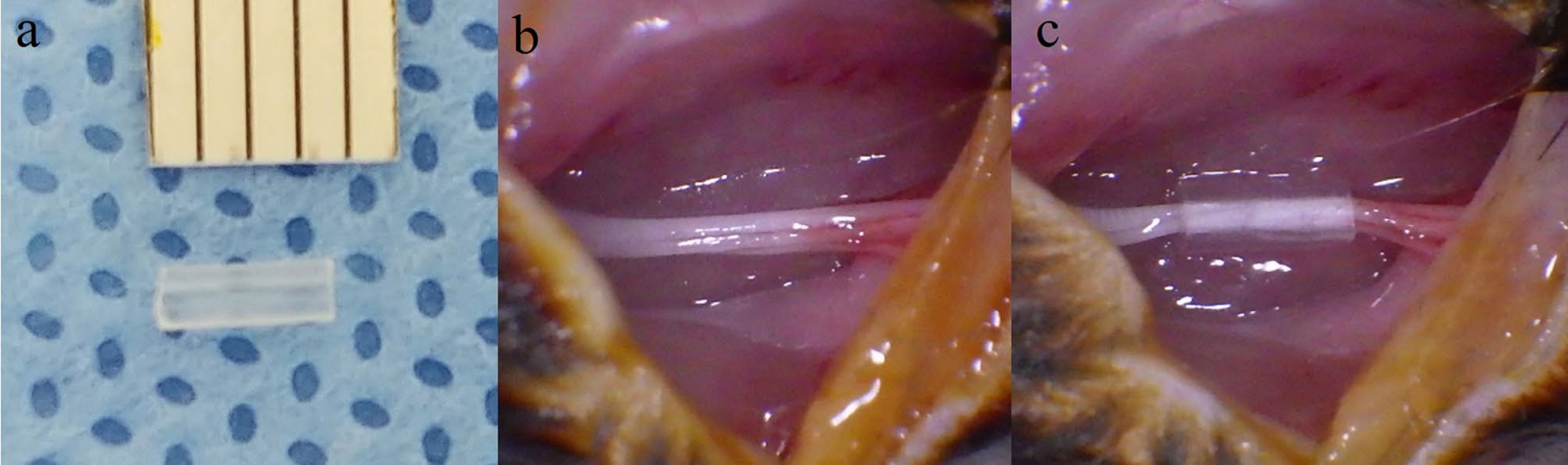
A murine model of compression neuropathy. A 3-mm length of a biologically inert silicone tube (**a**, inner diameter 0.4 mm) is placed around the sciatic nerve (**b**), providing a model of compressive neuropathy (**c**).

The mice were anesthetized with isoflurane-mediated inhalation anesthesia, and the right hindquarters were shaved, washed with 70% ethanol, prepped with povidone iodine, and a trans-gluteal approach allowed sciatic nerve exposure. To create a murine model of compressive neuropathy, a sterile 3-mm long biologically inert silicone tube with an inner diameter of 0.4 mm atraumatically encircled the sciatic nerve proximal to the trifurcation site^27–29^. (Fig. 5) The skin incision was closed using 4 or 5 interrupted 4-0 Prolene sutures (Ethicon, Somerville, NJ). The compressive tubes were left in place for a compressive phase of 6 weeks as a model of compression neuropathy, after which decompression surgery was performed to mitigate the compressive lesion^29^. The tube was removed atraumatically using micro-Jeweler forceps. All surgical procedures were performed by a single microsurgical technique-trained orthopedic surgeon using a sterile microsurgical technique under an operating microscope (OPMI pico, Carl Zeiss, Oberkochen, Germany). After the surgery, the mice were returned to their cages, allowed free activity, and were observed under the supervision of the attending veterinarian.

### Experimental design

**Figure 6:**
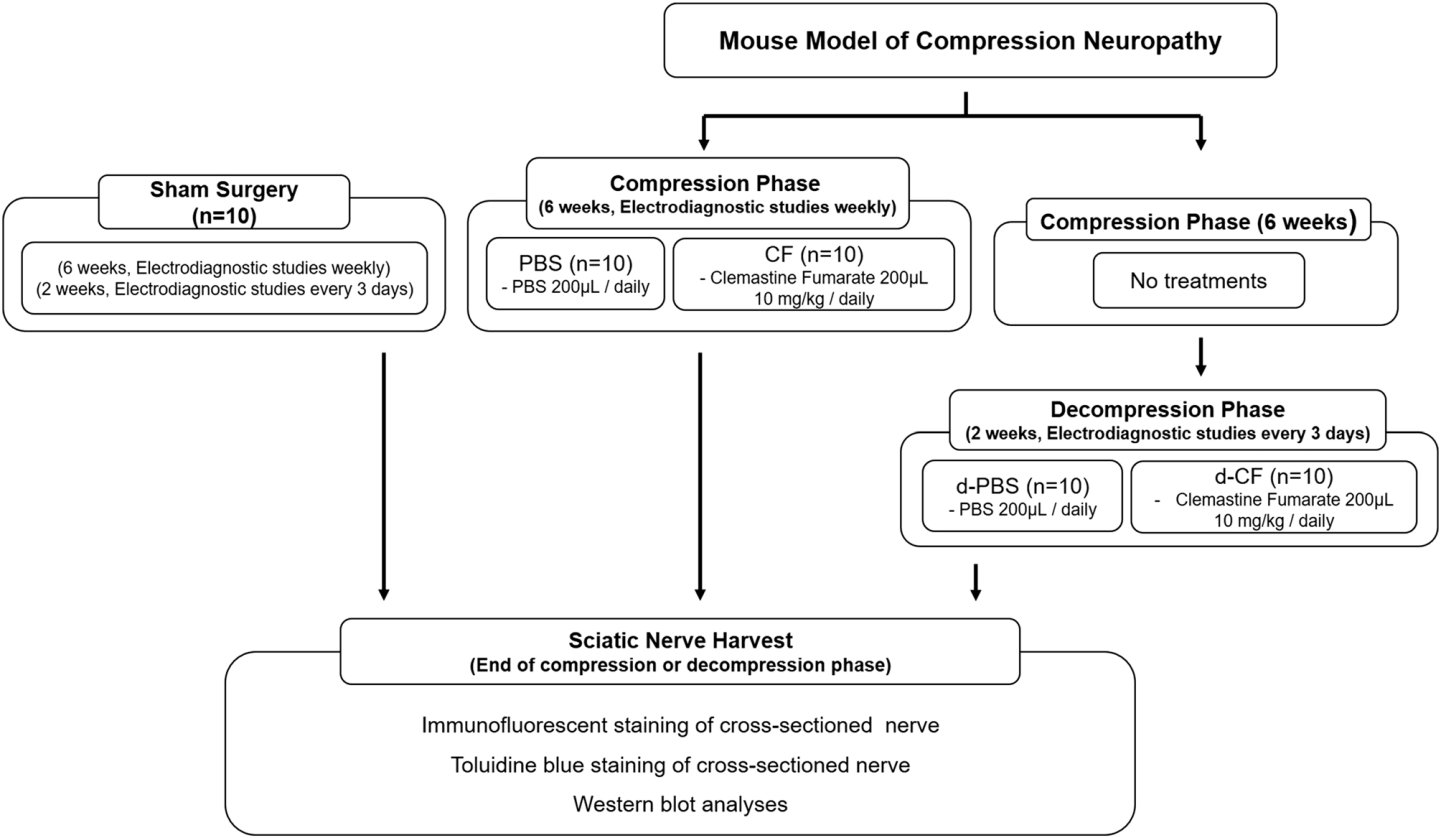
Scheme of experimental design. (PBS, control mice treated with phosphate buffered saline during compression phase; CF, mice treated with clemastine fumarate during compression phase; d-PBS, control mice treated with phosphate buffered saline after decompression phase; d-CF, mice treated with clemastine fumarate after decompression phase; n = 10/group).

Our murine model of compression neuropathy consists of two phases: compression and decompression. Mice underwent compressive surgery using a compressive tube and were assigned to one of the following groups (Fig. 6): (1) the PBS group, which received phosphate buffered saline (PBS) solution throughout the compression phase of 6 weeks and served as the untreated control in compression phase (n = 10); (2) the CF group, which received clemastine fumarate (CF; Cayman Chemical, Ann Arbor, MI) daily throughout the compressive phase (n = 10); (3) the d-PBS group, which received PBS throughout the decompression phase of 2 weeks after compression phase of 6 weeks without treatments and served as the untreated control in mice that underwent decompression surgery (n = 10); and (4) the d-CF group, which received clemastine fumarate daily throughout the decompression phase after compression phase without treatments (n = 10).

For treatment with clemastine fumarate, an active drug solubilized in sterile PBS solution was administered via intraperitoneal injection at a dose of 10 mg/kg, which was based on previous animal studies on the effect of CF on demyelinating disorders in CNS^5–8^. Electrodiagnostic studies were performed preoperatively and every week during the compression phase as well as every 3 days during the post-decompression phase starting day 1 post-decompression. For histomorphometric and Western blot analyses, mice from each group were euthanized at 6 weeks of compression or at 2 weeks after decompression, and the sciatic nerve was harvested. Three or four nerves from each group were used for analysis. The study was designed in accordance with ARRIVE guidelines.

### Electrodiagnostic studies

Electrodiagnostic studies to measure latency and amplitude were performed as in previous studies^12,13^. Latency and CMAP amplitudes were measured at the aforementioned time points. These data were gathered in vivo under isoflurane-mediated inhalation anesthesia using an electromyography machine (Medelec Synergy, Oxford instrument, UK)^30^. The recording needle electrode was placed into the tibial nerve-innervated calf muscle (gastrocnemius), and the stimulating needle electrode was placed at the proximal site of the sciatic notch. The distance between the recording and stimulating sites was kept constant using a 2 cm reference zig. The reference recording electrode was inserted into the dorsal aspect of the foot. The reference for the stimulating needle electrode was placed in the ipsilateral lumbar paraspinal muscle.

### Immunofluorescence analysis of cross sectioned nerve

The full length of the sciatic nerve was harvested after compression tube removal and fixed in 4% paraformaldehyde (PFA) at 4 °C overnight. The nerves were embedded in paraffin, and cross-sections were taken in the middle portions of the compression area. The 5 µm cross sections were taken from the embedded blocks and mounted on slides. The nerve sections were deparaffinized and serially rehydrated using xylene and ethanol. Antigen retrieval was performed using 0.01 M citrate buffer (pH 6.0), which was microwaved for 15 min. Permeabilization and blocking of nonspecific binding were performed with Tween 20 and 1:20 diluted goat serum (ab7481, Abcam, Cambridge, UK), respectively. Primary antibody staining was performed with anti- NF heavy chain antibody (1:500, ab4680; Abcam) and anti-P0 antibody (1:200, ab31851; Abcam) in 5% BSA. Incubation with 4′,6-diamidino-2-phenylindole (DAPI, 1:1000, ab228549; Abcam) and fluorescent secondary antibodies (Alexa Fluor 488, and 594-conjugated antibodies [1:500, ab197485 and ab150088, Abcam]) was performed after washing the sections in PBS to remove the primary antibodies. Fluorescent images were captured with a Leica DMI4000B fluorescence microscope (Leica Microsystems, Wetzlar, Germany) and semiautomatic analysis was performed with ImageJ (U.S. National Institutes of Health) to determine the number of NF- or P0 expressing axons.

### Histomorphometric analysis of the cross-sectioned nerve

Nerve segments were immersed in fixative solution (2% glutaraldehyde, 2% paraformaldehyde in 0.05 M sodium cacodylate buffer) overnight. After fixation, nerves were postfixed in 1% osmium tetroxide in 0.1 M PBS, treated with 0.5% uranyl acetate, and dehydrated using serial ethanol washes. Samples were serially incubated in 1:1 and 1:2 ethanol as well as Spurr’s resin, and then transferred to 100% Spurr’s resin. The specimens were transferred to Beem flat-embedding molds and baked at 70 °C overnight. Blocks were cut with an ultramicrotome to obtain 500-nm sections and stained with 1% toluidine blue. Images of cross-sectioned sciatic nerves were obtained using light microscopy and were analyzed using ImageJ software to determine axoglial diameter, axon diameter, myelin thickness and G ratio (axonal diameter/axoglial diameter on cross-section). Each parameter was measured using 50 randomly selected axons in each image, and all 300 axons per group were analyzed.

### Western blot analyses

Nerve segments were homogenized and extracted using RIPA lysis buffer containing protease/phosphatase inhibitor (GenDEPOT) and lysates were sonicated ten times for 30 s. The homogenized sample was centrifuged at 15,000 rpm for 10 min at 4 °C and the cleared lysates were quantified using a BCA protein assay kit (GenDEPOT). Equal amounts of protein were separated on 12% SDS-PAGE gels and transferred to a polyvinylidene fluoride (PVDF) membrane (Bio-Rad). Blots were probed with antibodies against P0 (1:1000, ab31851; Abcam) and MAG (1:1000, 34-6200; Invitrogen). β-actin (1:1000, 4967; Cell Signaling Technology) was used as a loading control. Protein expression was quantified using ImageJ software.

### Statistical analysis

All results are expressed as mean ± standard error of the mean. Statistical analysis was performed using GraphPad PRISM 9 (GraphPad Software, San Diego, CA) with *p* < 0.05. All data were tested for normality and lognormality. Two-way analysis of variance (ANOVA) with Turkey’s post-hoc test was performed to compare the means among three groups at each time points in electrodiagnostic data. One-way ANOVA with Turkey’s post-hoc test was performed in axonal counts and morphological analysis of peripheral nerve. Mann–Whitney U test was performed for comparisons of the means between two groups in Western blot analysis.

## Supplementary Information

Supplementary Figures.
