# Supplementary material for: Clemastine improves electrophysiologic and histomorphometric changes through promoting myelin repair in a murine model of compression neuropathy
Source: Sci Rep. 2021 Oct 22;11:20886. doi: 10.1038/s41598-021-00389-1 (PMC8536687; doi:10.1038/s41598-021-00389-1)
Supplement: Supplementary file 1 — Supplementary Figures. [file 41598_2021_389_MOESM1_ESM.pdf]

Supplementary Information

Clemastine improves electrophysiologic and histomorphometric changes through promoting myelin repair in a murine model of compression neuropathy

Jung Il Lee, M.D., Ph.D, Jong Woong Park, M.D, Ph.D, Kyung Jun Lee, B.S, Duk Hee Lee, M.D., Ph.D

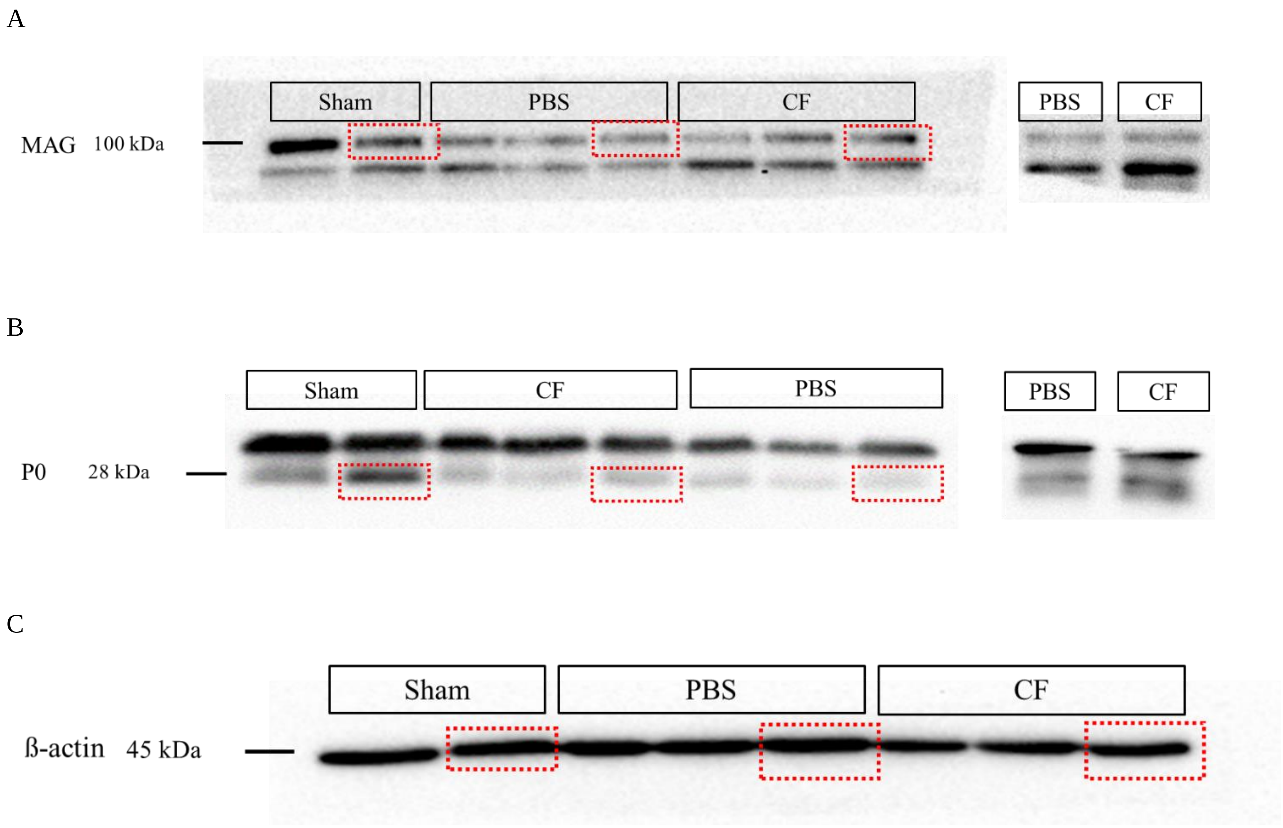

Supplementary Figure 1. Uncropped Western blot images displayed in compression phase of Figure 4 for expression of: myelin associated glycoprotein (MAG) (A); myelin protein 0 (P0) (B); ß-actin (C). The dotted rectangles delineate the areas shown in Fig. 4.

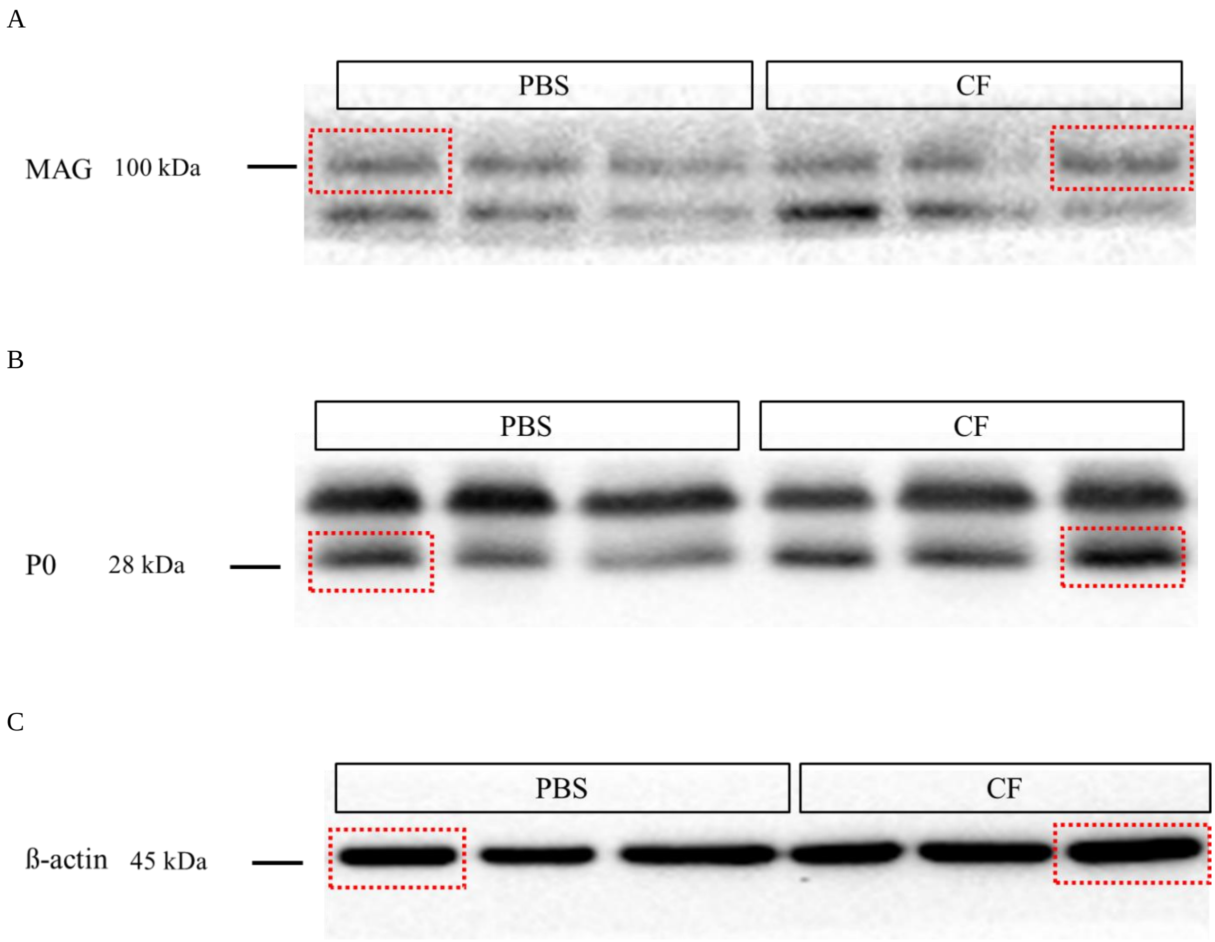

Supplementary Figure 2. Uncropped Western blot images displayed in decompression phase of Figure 4 for expression of: myelin associated glycoprotein (MAG) (A); myelin protein 0 (P0) (B); ß-actin (C). The dotted rectangles delineate the areas shown in Fig. 4.
